# Monitoring and radioecological characteristics of radiocesium in Japanese beef after the Fukushima nuclear accident

**DOI:** 10.1007/s10967-016-5021-0

**Published:** 2016-09-07

**Authors:** Georg Steinhauser

**Affiliations:** Institute of Radioecology and Radiation Protection, Leibniz Universität Hannover, 30419 Hannover, Germany

**Keywords:** ^137^Cs, Fukushima nuclear accident, Cattle, Rice straw, Food safety

## Abstract

After the Fukushima nuclear accident, beef proved to be a problematic food item with several exceedances entering the market. The reason was contaminated rice straw that was fed to cattle. Japanese authorities responded quickly to the exceedances and made beef one of the most-monitored food items after the Fukushima accident with more than a million samples within 5 years. Activity levels dropped quickly and are now considerably below the regulatory limit. No exceedance of the regulatory limit was observed after October 2012. The monitoring campaign focuses on the pre-market to catch any exceedances before they reach the consumer. The analysis also showed that some late beef samples exhibited an unusually high ^134^Cs/^137^Cs activity ratio. The reason for this is unknown.

**Electronic supplementary material:**

The online version of this article (doi:10.1007/s10967-016-5021-0) contains supplementary material, which is available to authorized users.

## Introduction

The Fukushima nuclear accident (March 11, 2011 in Fukushima prefecture, Japan) caused a widespread contamination of air, water, soil and food with mostly volatile, anthropogenic radionuclides, especially ^131^I (T_1/2_ = 8 days), ^134^Cs (T_1/2_ = 2 years) and ^137^Cs (T_1/2_ = 30 years). Contaminated food is the most important route for the intake of radionuclides [1–3] and possibly outperforms inhalation in terms of significance as an alternative route for the incorporation of radionuclides [4]. Therefore, monitoring of food and food-related research has been treated with high priority in the aftermath of the nuclear accident [5–11].

Japanese authorities were well aware of the importance of food control and launched a monitoring campaign that is unprecedented in human history. Studies using whole-body counters [12] or food-duplicates [10] have shown very low incorporation rates of the population through food in Japan; they were about three orders of magnitude lower than what was observed in the Chernobyl-affected areas in the first year after the Chernobyl nuclear accident [13]. In a previous study [14], we assessed the effectiveness of the food monitoring campaign by means of statistical methods. We could show that for most food items, the monitoring proved to be exceptionally effective, the only major exception being beef (cattle meat). In the present study, we therefore shall discuss in depth the issue of beef contamination, monitoring of beef and radioecological characteristics that can be derived from the data.

## Materials and methods

The data used in this study were reported online by the Ministry of Health, Labor and Welfare (MHLW) [15]. The data are reported on this website [15] for various food types in various intervals. This study collectively includes all data that were reported for “cattle meat” for 60 months after the Fukushima nuclear accident (i.e. from Mach 2011 until March 2016). It is important to note that the data were sorted according to the date of the release (“press release”) of the respective results. Since for radioecological considerations the date of sampling is much more relevant than the date of reporting, the data in the data base were re-sorted to the date of sampling. For some samples, no date of sampling was given in the data base. In such cases, the respective dates of measurement were taken instead. A very small number of reports did not even include a date of measurement; in this case, the date of reporting was used instead.

Measurements conducted by the Japanese authorities employed mostly NaI detectors, but sometimes also HPGe detectors. Later in the monitoring campaign, also CsI detectors were used in increasing numbers.

Further it is important to note that the vast majority of data were below the detection limit. The detection limits varied from measurement to measurement, but initially they were typically below <50 Bq/kg for the sum activities of ^134^Cs and ^137^Cs. Later, as activity concentrations decreased, detection limits also got lower and lower (<25 Bq/kg). For discussions where trends in the radiocesium activity concentrations are observed, only “positive” measurements, i.e. measurements that exceeded the detection limit, were collected and used. In this particular case, the discussion of data that are below the detection limit was deemed pointless. For other discussions where the number of measurements is relevant (irrespective of the result), naturally the entire number of measurements (including non-detectables) was collected.

The data base generally further distinguishes between samples taken in the “pre-market” (also termed “not marketed” samples) and taken in the “post-market” (“marketed” samples). Pre-market samples were taken directly at the producing facility and thus were not taken in competition between food monitoring officers with consumers. Post-market samples, in contrast, were taken at the market in competition with consumers. These are important characteristics for the assessment of the effectiveness of the food monitoring campaign, see [14]. Only in the first months after the accident, a (partly considerable) fraction of the samples was taken without any specification into pre- or post-market (“not specified”).

## Results and discussion

Monitoring of radionuclides in food was identified by the Japanese authorities as a significant factor in securing public health following the Fukushima nuclear accident. As outlined previously [14], the monitoring of beef, however, started with delay, because beef was given only second priority in the early aftermath of the accident. In any case, after exceedances in the category “beef” were spotted in the post-market, beef was then measured with high priority of at least 10,000 samples monthly. It has shown that contaminated rice straw from Fukushima prefecture was fed to cattle causing high contamination levels in cattle even in remote locations. Table S1 (Supporting Information) summarizes all the samples which exceeded the limits and which MHLW attributed to the possible distribution and feeding of contaminated rice straw. The entity of data provided by MHLW is also available in pdf form from the MHLW website [15].

**Fig. 1:**
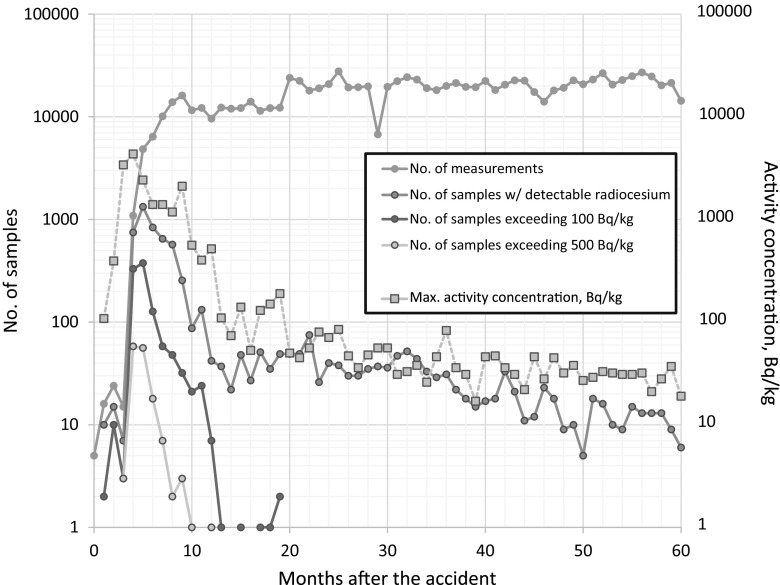
Characteristics of the beef monitoring campaign in absolute numbers on a monthly basis and the maximum activity concentration found in the respective month (in Bq/kg)

**Fig. 2:**
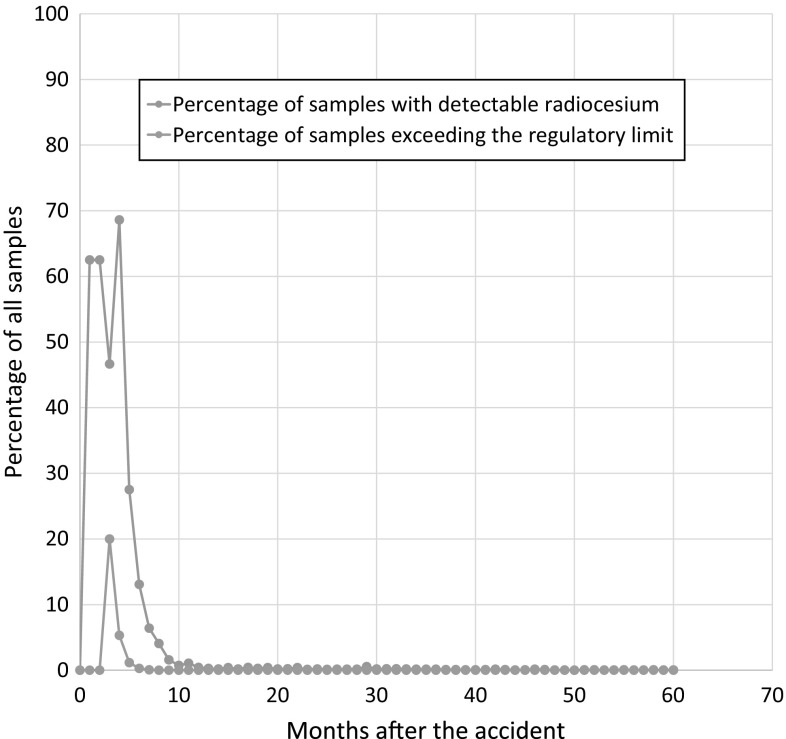
Percentage of samples of beef exceeding the detection limit and regulatory limit, respectively. Please note that the regulatory limit of radiocesium in foods including beef was lowered as of April 1, 2012 from 500 to 100 Bq/kg

The latest exceedance of the regulatory limit of a cattle meat sample was observed in a beef sample from Gunma prefecture taken on October 25, 2012, with a total radiocesium activity concentration of 190 Bq/kg. Although since October 2012 no more exceedances of the regulatory limit were found in beef, the authorities have continued until the present day with this very high monitoring numbers that correspond to the staggering number of several hundred of samples to be taken and measured per day. Figure 1 shows a summary of the main characteristics of the monitoring campaign of beef. Please note that the regulatory limits were 500 Bq/kg from March 2011 until the end of March 2012; from then on, the regulatory limit was set to 100 Bq/kg [16]. Since November 2012, the maximum activity concentrations have fallen to several tens of Bq per kg, with a clear downward trend. Please note that only a small fraction of about ten samples of about 20,000 samples taken each month presently even exhibit a detectable radiocesium activity concentration. The vast majority of samples passes the screening without even exceeding the detection limit. The relative fraction of samples exceeding the detection and regulatory limit, respectively, is shown in Fig. 2. It can be seen that about a year after the accident, the fraction of samples exhibiting detectable activities decreased dramatically.

Figure 1 also shows that the maximum activity concentration exhibited a spike, with the number of samples taken skyrocketing about a month or two later. This shows the rapid response of the Japanese authorities. They might have reacted faster if the early exceedances had been spotted earlier by faster measurement. The first exceedance in cattle meat in fact was observed in a sample that was taken on June 10, 2011, but this sample was not measured before July 11, 2011. Once the data of this exceedance were on the table, Japanese authorities reacted quickly and increased the number of measurements drastically.

**Fig. 3:**
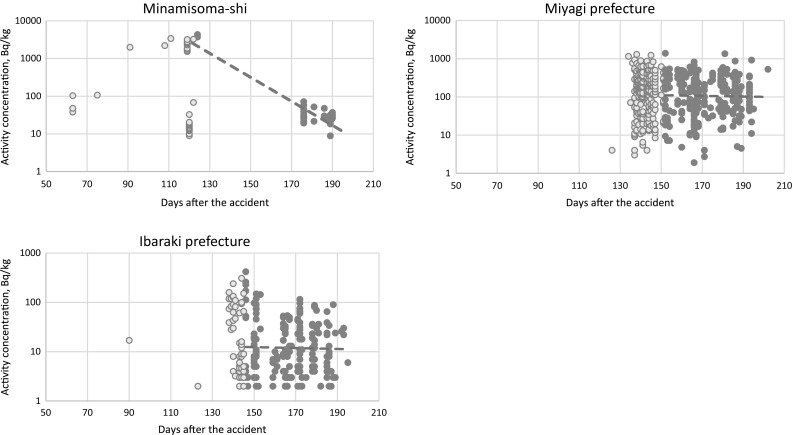
Decline characteristics of radiocesium activity concentrations (sum of ^134^Cs + ^137^Cs) in beef in three locations around Fukushima Daiichi NPP for the estimation of effective half-lives of radiocesium in beef

As it becomes obvious, that the activity levels in beef dropped rather quickly, we tried to shed some light onto the question of the decline characteristics of radiocesium in food. For this purpose, not the entity of samples can be taken as the sampling intensity varied from prefecture to prefecture and municipality to municipality. For the purpose of this investigation, three areas with a rather high monitoring density were chosen: (1) Minamisoma-shi municipality inside Fukushima prefecture. This municipality is located north of the reactor with a relatively low contamination level, although it had been affected by late dust outbreaks from the Fukushima Daiichi NPP site [17]. (2) Miyagi prefecture in the north of Fukushima prefecture and (3) Ibaraki prefecture south of Fukushima prefecture. The decay characteristics are shown in Fig. 3. Activity levels in living organisms are influenced by accumulation processes (intake by ingestion and possibly inhalation) and diminishing processes (excretion and physical decay of the radionuclides). These factors influence the final activity level observed in the organism. In the early phase after a sudden exposure to radionuclides (especially radiocesium), accumulation processes will be the dominating factor leading to constantly increasing activity levels until a maximum is reached. After that maximum has been reached, excretion of radiocesium will dominate and outweigh further accumulation by contaminated pasture, thus leading to decreasing activity levels. This downward trend in activity exhibits an exponential decline characteristics that can be used to calculate effective half-lives.

Following this general assumption, also radiocesium in beef proved to exhibit an upward trend in the early phase (light blue data points in Fig. 3). It was decided to use data for the calculation of the effective half-life beginning at the date when the maximum value was reached (dark blue data points). This approach of disregarding increasing activity levels had been proven reasonable in the past [18, 19]. Only data indicating detectable radiocesium levels were used for calculating the effective half-life. As an arbitrary end date, September 2011 was chosen, when still a considerable number of samples exhibited detectable concentrations. At a later point, single detections would have received disproportionally large weight. The decay constant λ of the effective half-life was obtained by calculating an exponential trend line of the decreasing activity concentration data points that was calculated using MS Excel. The effective half-lives (derived from λ) of radiocesium (sum of ^134^Cs + ^137^Cs) were 9 days in Minamisoma, 350 days in Miyagi prefecture, and 327 in Ibaraki prefecture. It is clear that these numbers are affected by many biases (such as governmental actions in the agricultural procedures), but at least they give a rough estimate of the extreme values observed when studying the decline characteristics of radiocesium in beef. In this particular case, it is interesting to note that the effective half-life exhibits such a large range (from a few days to almost 1 year). Possible reasons for this unexpected observation are different data density in the observed regions as well as actively influencing factors such as the governmental efforts in managing food safety. In any case, it is obvious that the late sporadic releases from the Fukushima Daiichi NPP site (resuspension of highly contaminated dust) that was observed in the Minamisoma area [17], did not show in the activity levels of beef (as it would have flattened the steep decay line in Fig. 3).

**Fig. 4:**
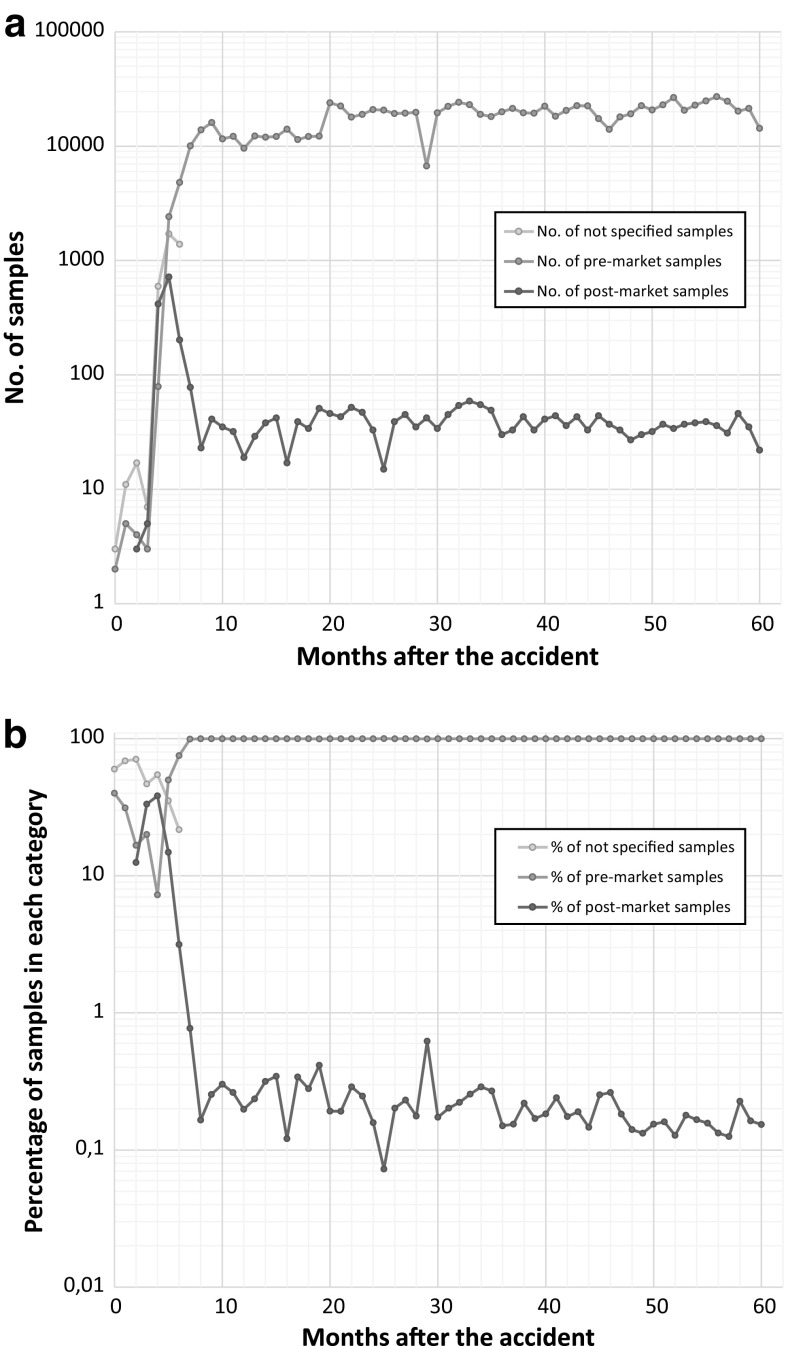
Pre- and post-market sampling of beef in absolute numbers (**a**) and relative numbers (percent) (**b**)

As stated above, the MHLW data base also distinguishes between pre- and post-market samples. In our previous publication [14], we showed that the discussion of pre- and post-market samples can allow assessing the effectiveness of the monitoring campaign. We had shown that the monitoring was effective for all food categories, with the exception of beef and tea. Figure 4 shows the distribution between pre-market and post-market beef samples (as well as not-specified-samples in the early phase), both in absolute numbers (Fig. 4a) and in percentages (Fig. 4b). It is obvious that, after the initial sampling phase (up to mid-2011), the main focus has been laid on pre-market sampling. However still roughly 30–50 samples are still taken in the post-market each month. We assume this relatively constant number of samples in the post-market has been chosen for quality control purposes to check if the beef in the post-market exhibits acceptable activity levels. Only in the beginning (i.e., summer 2011), the number and percentage of post-market samples was significantly higher: This was the time with the most exceedances. In a clear response to these exceedances, the focus was shifted towards post-market samples. That was the time, when the post-market had to be cleared from above-limit beef that was already available to the consumers.

**Fig. 5:**
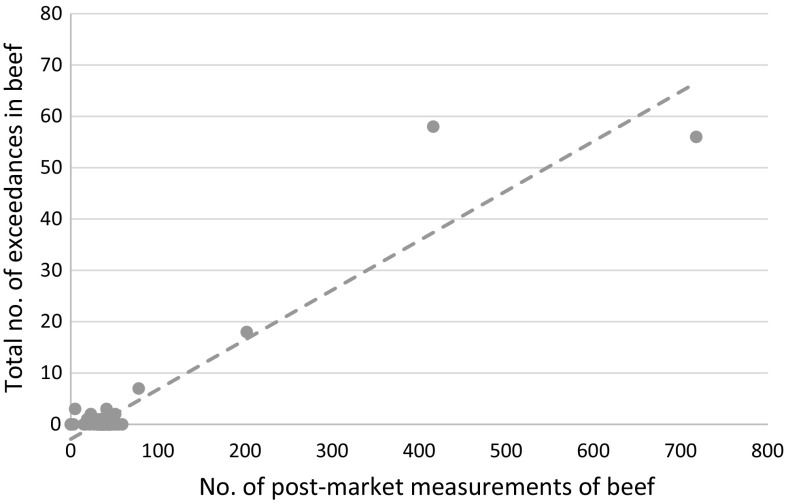
Correlation between post-market sampling and the number of exceedances in a certain month

The correlation between post-market sampling and the number of exceedances in a certain month during the entire period of observation (March 2011–2016) is shown in Fig. 5 (based on the entity of beef samples in the MHLW data base with a linear trend line). This graph makes it obvious that the focus on increased post-market sampling was clearly correlated with the number of exceedances observed in the same month. The more exceedances are observed, the more samples are taken in the post-market to minimize the risk of consumers purchasing and consuming above-limit beef.

**Fig. 6:**
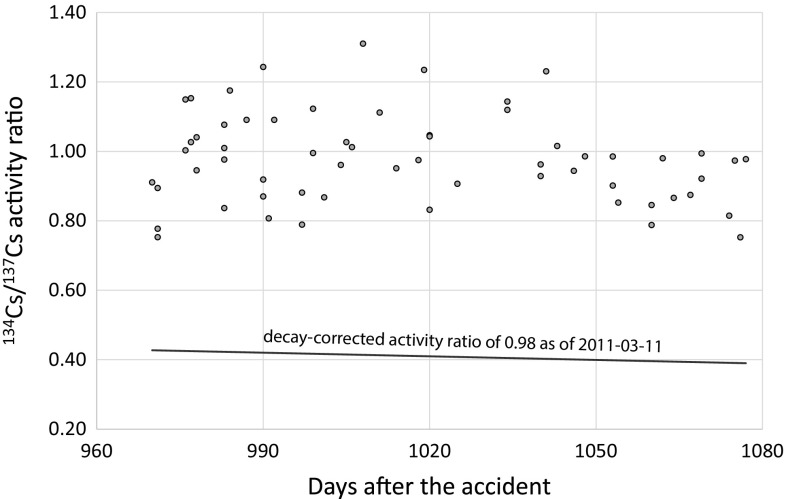
A selection of data that were reported unusually long after sampling and measurement. They all exhibit a clearly higher ^134^Cs/^137^Cs activity ratio than expected from the decay corrected value of 0.98 that is typical for Fukushima’s integral releases [16]

During the investigation of the data set, we observed that a limited number of samples taken in 2013 and early 2014 were reported several months later in 2014. It is unclear whether or not this is a result of a typo or human mistake or whether this reporting was intentionally delayed. The respective samples exhibited undetectable or very low activity concentrations and were also numerically far in the sub-percent range. Therefore, this side note should not fuel any speculations on political intentions or any type of conspiracies causing this delay in reporting. As an example, Fig. 6 shows the data reported on April 24, 2014 that were sampled between November 2013 and February 2014 (for these selected data see Supporting Information, Table 2). These samples were also measured on the same day of sampling; only the press release date was delayed by months which appears rather unusual. All of these measurements shown in Fig. 6 were measured on an HPGe detector. In any case, when looking at these “delayed” data, it becomes obvious that they all exhibited a very unusual, namely unusually high ^134^Cs/^137^Cs activity ratio. It is possible that the authorities went after possible explanations of this very unusual characteristics before they decided to report the data to the public. A higher-than expected ^134^Cs/^137^Cs activity ratio may be indicative of late criticalities and continuing releases of radiocesium in the environment, which, however, can be excluded in the present case as the data in question stemmed from 2013 to 2014 (at a time when the reactor cores were back under control). It is unclear what the reason of the higher activity ratio in these samples is. It is also possible that these deviations are only the result of higher counting uncertainties (please note that we do not have individual uncertainties or measurement conditions such as the duration for the individual measurements). In any case, it is an eye-catching fact that these data only originate from three prefectures, namely Hokkaido, Tokushima, and Miyagi.

## Conclusions

In the aftermath of the Fukushima nuclear accident, beef proved to be a problematic food item with several above-limit items entering the market. The reason for the high contamination level is contaminated rice straw that was fed to cattle. After the monitoring of beef started with some weeks of delay, the Japanese authorities responded quickly to the exceedances and made beef one of the most-monitored food items after the Fukushima nuclear accident. Activity levels dropped quickly and are now considerably below the regulatory limit. The effective half-lives observed for radiocesium in beef in various regions are subject to strong fluctuations (from a couple of days to almost a year). Japanese beef has now become one of the safest food items available in Japan. The monitoring focuses on the pre-market to catch any exceedances before they reach the consumer. Only when a large number of exceedances was observed for beef, the monitoring expanded to the post-market to catch food items that have already reached the markets. Today’s high level of food safety is illustrated by the fact that despite high numbers of samples (>10,000 per month) being measured, no exceedance was observed in the category “cattle meat” after October 2012 (19 months after the accident). These scientific facts strongly contradict current media reports [20] which suggest that local food in Japan was not safe to eat. The opposite is the case.

During the analysis of more than a million beef samples we also found a couple of samples that exhibited an unusually high ^134^Cs/^137^Cs activity ratio and that were published with delay. The reason for this phenomenon and the reasoning for the delayed publication of these peculiar data remains unknown and unexplained to the author.

## Electronic supplementary material

Below is the link to the electronic supplementary material.

Supplementary material 1 (XLSX 16 kb). Table S1. List of beef samples exceeding the limits after cattle may have been fed with contaminated rice straw (a) from Fukushima prefecture and (b) from other prefectures

Supplementary material 2 (XLSX 18 kb). Table S2. Selected data of beef with an unusually high ^134^Cs/^137^Cs activity ratio which were published with significant delay on April 24, 2014
